# High-Resolution Labeling and Functional Manipulation of Specific Neuron Types in Mouse Brain by Cre-Activated Viral Gene Expression

**DOI:** 10.1371/journal.pone.0002005

**Published:** 2008-04-16

**Authors:** Sandra J. Kuhlman, Z. Josh Huang

**Affiliations:** Cold Spring Harbor Laboratory, Cold Spring Harbor, New York, United States of America; University of Washington, United States of America

## Abstract

We describe a method that combines Cre-recombinase knockin mice and viral-mediated gene transfer to genetically label and functionally manipulate specific neuron types in the mouse brain. We engineered adeno-associated viruses (AAVs) that express GFP, dsRedExpress, or channelrhodopsin (ChR2) upon Cre/loxP recombination-mediated removal of a transcription-translation STOP cassette. Fluorescent labeling was sufficient to visualize neuronal structures with synaptic resolution in vivo, and ChR2 expression allowed light activation of neuronal spiking. The structural dynamics of a specific class of neocortical neuron, the parvalbumin-containing (Pv) fast-spiking GABAergic interneuron, was monitored over the course of a week. We found that although the majority of Pv axonal boutons were stable in young adults, bouton additions and subtractions on axonal shafts were readily observed at a rate of 10.10% and 9.47%, respectively, over 7 days. Our results indicate that Pv inhibitory circuits maintain the potential for structural re-wiring in post-adolescent cortex. With the generation of an increasing number of Cre knockin mice and because viral transfection can be delivered to defined brain regions at defined developmental stages, this strategy represents a general method to systematically visualize the structure and manipulate the function of different cell types in the mouse brain.

## Introduction

Neuronal circuits consist of diverse cell types, and there is increasing evidence that each cell type often displays stereotyped connectivity and carries out specialized functions. To understand the organization and operation of neuronal circuits, it is therefore necessary to be able to visualize the structure and connectivity of different cell types at high resolution and to manipulate the function of specific cell types with precision. Of particular relevance are the GABAergic inhibitory circuits in the neocortex. GABAergic inhibition is crucial in all aspects of neural circuit operation in the cortex and is mediated by diverse interneuron cell types. Because different cell types are highly intermingled and even neighboring neurons differ in their connectivity and function [1]–[3], such heterogeneity and complexity has been difficult to penetrate by conventional anatomical and physiological techniques. For example, there is increasing evidence that GABAergic synapses are structurally modified by sensory experience and neural activity [4]–[6], potentially leading to significant reconfiguration of neural circuits. However, there has been no study that examines the structural dynamics of defined classes of cortical inhibitory neurons and synapses in the intact brain. This gap in knowledge is largely due to the heterogeneity of cortical GABAergic cell types and the lack of a high resolution labeling method.

Genetic strategies can significantly contribute to studying GABAergic circuits and neural circuits in general because they tap into the intrinsic gene regulatory mechanisms that generate and maintain the cellular diversity of the nervous system [7]. Because different cell types often display distinct gene expression profiles [8]–[10], transcriptional promoters provide genetic access to visualize and manipulate different cell types. Gene knockin and transgenesis using bacterial artificial chromosomes (BAC; [11]) are two useful techniques to introduce exogenous genes into a cell type of interest defined by the expression of an endogenous gene. In particular, Cre/loxP recombination-regulated gene expression is an efficient and powerful approach to systematically label and manipulate defined cell types [12]. This binary gene expression strategy involves the combination of two mouse strains: a “driver” strain expressing Cre-recombinase in specific cell types and/or brain regions, and an “indicator” strain capable of expressing a gene(s) of interest upon Cre/loxP recombination. To date, an increasing number of cell-type restricted driver lines have been generated [12]. However, the current implementation of this strategy suffers three major shortcomings. First, the spatial and temporal expression pattern of any one single gene may not be ideal to manipulate a cell type at a restricted developmental stage and brain region. Second, with only a few exceptions, the expression level of fluorescent markers introduced by either knockin or BAC trangenics are often insufficient to label fine neuronal structures such as neuronal axons and synapses; expression levels from available indicator lines are orders of magnitude lower than what is necessary for high resolution imaging in vivo. Third, mouse genetic engineering, especially when involving multiple strains, is time consuming and costly.

Viral-mediated gene delivery represents an alternative and powerful strategy to label and manipulate neurons in the mammalian brain. Because of their multi-copy transfection of a single neuron and the use of strong and ubiquitous transcription promoters, viral-mediated delivery can often achieve high-level gene expression and thus bright labeling of fine structures such as neuronal synapses [13]–[15]. In addition, viral transfection can be targeted to specific brain regions and developmental stages by stereotactic injection [16]. Furthermore, neurotrophic viruses suitable for longitudinal studies have been well characterized and can now be efficiently engineered at low cost [17]. However, a major drawback of viral-mediated gene delivery is the lack of cell-type specificity - currently there is no general strategy to restrict viral-mediated gene expression to defined cell types for a prolonged time period. Here we describe a method that combines Cre-recombinase knockin mice and Cre-activated adeno-associated viral vectors to achieve high-level, stable, and cell-type specific gene expression. This method is simple, highly efficient, and allows chronic live imaging of defined classes of synapses in vivo and light activation of neuronal spiking. With the establishment of increasing number of Cre knockin and transgenic lines (Cre drivers), this method represents a general strategy to systematically visualize and manipulate specific cell types in-vivo.

Using this method to label a specific class of inhibitory cell, the neocortical parvalbumin (Pv) inhibitory interneuron, we were able to chronically image, for the first time, the fine axonal structures and dynamics of Pv inhibitory axon boutons in vivo. We found that although the majority of putative Pv presynaptic boutons were stable in young adult mice, bouton additions and subtractions on axonal shafts were readily observed at a rate of 10.10% and 9.47%, respectively, over 7 days. Our results indicate that Pv inhibitory circuits maintain the potential for structural re-wiring in post-adolescent cortex.

## Results

### Cre-activated adeno-associated viral vectors confer cell-type specific and high-level gene expression in the neocortex

**Figure 1:**
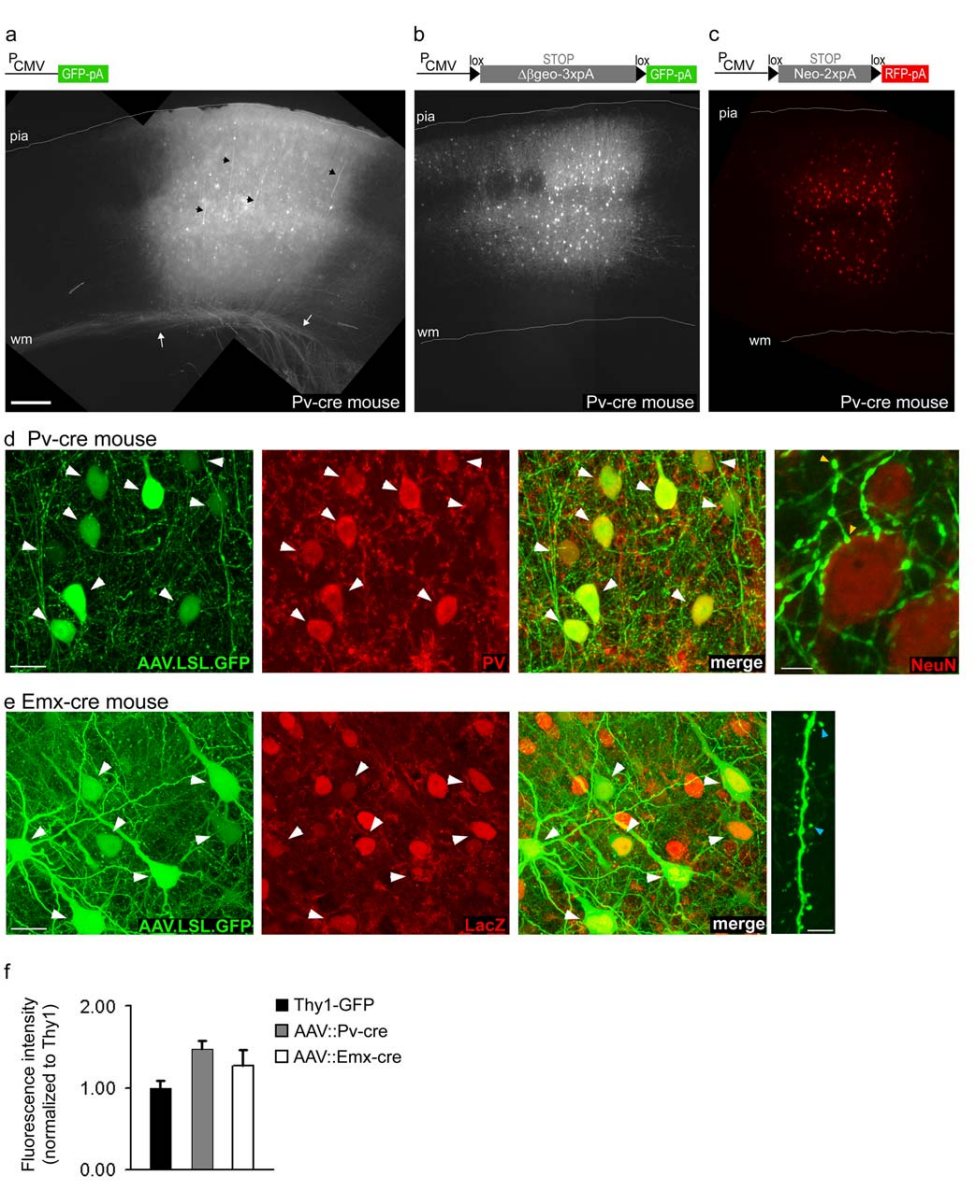
Cre-activated AAV vectors confer cell-type specific and high-level gene expression in brains of Cre knockin mice. (a) Injection of a generic AAV-GFP virus into neocortex of a Pv-cre mouse labeled neurons non-specifically. Pyramids can be recognized by the presence of thick apical dendrites (black arrowheads) and projecting axons (white arrows). (b) Injection of the AAV-lox-STOP-lox (LS_1_L)-GFP virus into the neocortex of a Pv-cre mouse specifically labeled neurons expressing Cre-recombinase. The STOP cassette is shown in gray in the schematic and is described in detail in Methods. Δβgeo-3xpA, a modified beta-galactosidae/neomycin STOP cassette with 3 poly-adenylation sites. (c) Injection of the AAV-LS_2_L-RFP virus into the neocortex of a Pv-cre mouse specifically labeled neurons expressing Cre-recombinase. A different version of STOP cassette is used here, Neo-2xpA, a neomycin STOP cassette with 2 poly-adenylation sites. P_CMV_, CMV promoter and β-globin intron; scale bar, 250 µm for a–c. (d) Co-localization (white arrowheads) of GFP and parvalbumin (PV) in neocortical basket interneurons in neocortex of a Pv-cre mouse injected with AAV-LS_1_L-GFP; scale bar, 20 µm. Far right, high resolution image of basket cell axons with “basket-like” terminal branches and boutons (yellow arrowheads) around pyramidal cell somata (labeled with NeuN immunofluorescence) characteristic to PV+ interneurons; scale bar, 5 µm. (e) Co-localization of GFP and LacZ in neocortical pyramidal neurons in a Emx-cre-nlsLacZ mouse injected with AAV-LS_1_L-GFP; scale bar, 20 µm. Note that not all LacZ^+^ pyramidal neurons were positive for GFP. Far right, spines (blue arrowheads) along a pyramidal cell apical dendrite; scale bar, 5 µm. (f) Quantification of GFP fluorescence intensity at the soma normalized to intensity of GFP-labeled pyramidal soma in Thy1-GFP mice; n = 15 cells for each group. The intensity range for both Thy1-GFP and AAV-labeled somata was large, only the brightest cells were selected for this particular comparison (see Methods for details). Thy1-GFP: 1+/−0.08; AAV-LS_1_L-GFP::Pv-cre: 1.5+/−0.1; AAV-LS_1_L-GFP:: Emx-cre 1.3+/−0.2). All AAV-injected tissue was fixed 12–15 days post injection of 1.5–3 month-old mice.

We chose to explore adeno-associated virus (AAV) because it is considered to be non-pathogenic and non-toxic to neurons [18]–[21], and easy to engineer. Here we show that a generic serotype 2/9 AAV-GFP virus efficiently transfected neurons in the neocortex and drove green fluorescent protein (GFP) expression in both glutamatergic pyramidal neurons and GABAergic interneurons by a strong and ubiquitous CMV promoter, *P_CMV_* (Fig. 1a, and data not shown). To render GFP expression conditional upon Cre-mediated recombination, we added a loxP-STOP-loxP (LSL) cassette between the CMV promoter and either GFP or red fluorescent protein (RFP) cDNA (Fig. 1b,c). STOP is a transcriptional and translational stop cassette containing multiple poly-adenylation signals. We constructed two different versions of STOP (see Methods for details): Δβgeo-3xpA (S_1_) is a shortened version of a beta-galactosidae/neomycin STOP cassette containing 3 poly-adenylation sites with proven functionality [22], and Neo-2xpA (S_2_) is a neomycin cDNA followed by 2 poly-adenylation sites. We then tested these “conditional AAVs” (AAV-LS_1_L-GFP; AAV-LS_2_L-RFP) using two different Cre knockin drivers, the Pv-cre mouse, which expresses Cre in parvalbumin-containing (PV^+^) GABAergic neurons [23], and the Emx-cre mouse, which expresses Cre in glutamatergic neurons in the forebrain [24]. Both versions of the LSL performed well.

**Figure 2:**
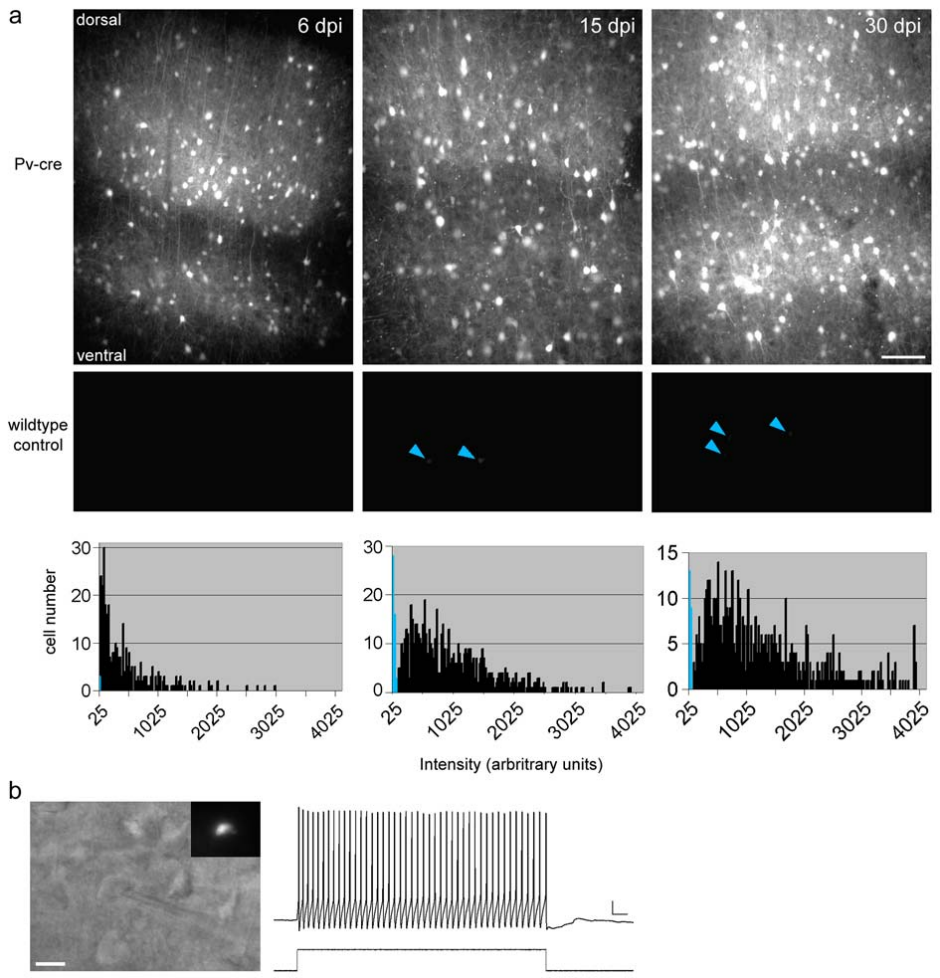
GFP expression in Pv-cre mice injected with AAV-LS_1_L-GFP is stable for months. (a) Pv-cre mice (top row; n = 3 mice for each time point) and wildtype control mice (middle row; n = 3 mice for each time point) were injected with AAV-LS_1_L-GFP and were perfused at 6,15,30, and 60 days post-injection (dpi). See Figure S3 for 60 dpi. All epi-fluorescence images were acquired with a CCD camera using the same acquisition parameters. The maximum exposure time that did not lead to saturation of signal was used (75 ms), and the same look-up table was applied to all of the images shown. GFP expression levels increased with time in Pv-cre mice. Under these conditions, sparse weak signals were detected in some wildtype control mice (blue arrowheads); scale bar, 100 µm. Bottom row, quantification of GFP intensity at somata of labeled neurons plotted for wildtype control mice (blue) and Pv-cre mice (black). Average number of GFP+ cells counted per animal in Pv-cre injected animals was (6,15,30 dpi): 110+/−34, 215.3+/−34, 195.3+/−26 cells. (b) Whole cell current clamp recording of an AAV-LS_1_L-GFP-transfected neuron from a 2.5 month-old Pv-cre mouse at 20 dpi. Left, DIC image of recorded cell; inset shows GFP fluorescence; scale bar, 10 µm. Right, example trace of spikes in response to a 250 pA current step; scale bar, 10 mV, 25 ms.

**Figure 3:**
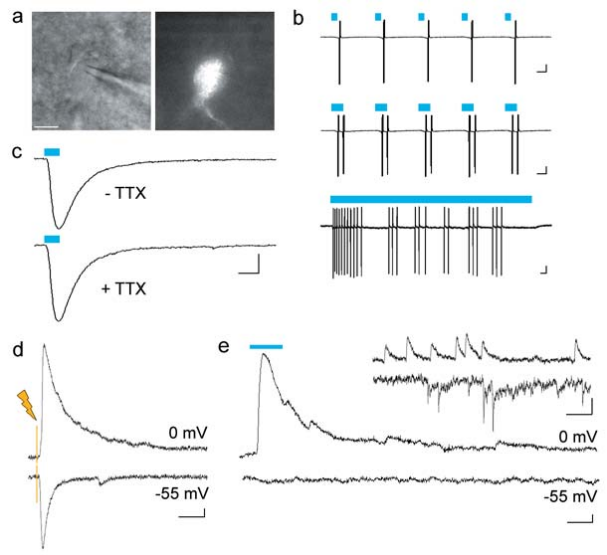
Functional demonstration that the Cre-activated AAV vector is cell-type specific. Pv-cre mice were injected with AAV-LS_2_L-ChR2mCherry and acute slices for electrophysiological recording were prepared. (a–c) Light stimulation directly elicited depolarizing responses in ChR2-positive neurons. DIC and fluorescent images of a ChR2-positive cell targeted for recording (a); scale bar: 10 µm. Spike responses to light stimulation of increasing duration (7, 14, and 500 ms as indicated by blue line) were recorded in cell-attached mode (b); scale bars: 40 pA, 25 ms. ChR2-mediated current was maintained in the presence of TTX. (c); scale bar: 50 pA, 10 ms. (d) Electrical field stimulation evoked both inhibitory post-synaptic current (upper trace, holding potential set to the reversal potential for AMPA/NMDA-mediated current, 0 mV) and excitatory post-synaptic current (lower trace, holding potential set to the reversal potential for GABA-mediated current, −55 mV), recorded in a pyramidal cell in whole-cell voltage-clamp mode; scale bar: 40 pA, 20 ms. (e) Light stimulation specifically evoked inhibitory post-synaptic current (upper trace), no excitatory post-synaptic current was detected (lower trace); scale bar: 20 pA, 20 ms. Inset, both inhibitory and excitatory spontaneous synaptic events were detected using the same recording conditions; scale bar: 20 pA, 50 ms.

In the neocortex, PV is expressed in a prominent class of GABAergic inhibitory interneurons, the basket interneuron, which are fast spiking and innervate the soma and proximal dendrites of pyramidal neurons. In the neocortex of Pv-cre mice injected with AAV-LS_1_L-GFP (single-tract injections, see Methods for details), GFP expression was almost exclusively restricted to PV^+^ interneurons (Fig. 1d). In a total volume of 1.46 mm^3^ examined from 4 animals, 97% of the GFP-expressing neurons (n = 416 GFP^+^ neurons) were also PV immuno-positive, indicating a high degree of cell-type specificity. In the same volume, a total of 479 PV^+^ neurons were identified, indicating an 86% average labeling efficiency by AAV-LS_1_L-GFP. High-level GFP expression allowed bright and spectacular labeling of exuberant basket cell axon arbors (Fig. 1d; Fig. 2a), including their highly characteristic local branches and perisomatic boutons around pyramidal cell somata (Fig. 1d; Fig. 3e). The LS_2_L gave a very similar labeling pattern (Fig. 1c and Fig. S1).

The labeling efficiency of AAV-LS_1_L-GFP varied according to distance from injection site: efficiencies of >96% (200 µm^3^ volume) were observed close to the center of injection, while at the edges of injection labeling was sparse enough so that the axons and dendrites of individual basket cells could be followed with high resolution (Fig. 3d–h). Furthermore, smaller volumes of injection lead to sparser labeling (data not shown). To achieve high efficiency labeling of basket cells in volumes greater than 200 µm^3^, multi-tract injections can be employed [25].

Emx-cre mice express Cre-recombinase and a nuclear-targeted lacZ in most if not all neocortical pyramidal neurons [24]. In the cortex of Emx-cre mice injected with AAV-LS_1_L-GFP, GFP expression was restricted to lacZ^+^ pyramidal neurons (Fig. 1e). In a total volume of 0.822 mm^3^ (n = 4 animals), 98% of GFP+ cells co-localized with LacZ. Labeling efficiency reached levels as high as 86% (0.51 mm^3^ volume). The characteristic apical and basal dendrites of layer 5 pyramidal neurons were readily apparent; dendrites were highly decorated with spines (Fig. 1e).

Several Thy1-GFP and -YFP transgenic lines have been successfully used to image structural dynamics in glutamatergic pyramidal neurons in-vivo [26]–[28], largely because the fluorescent signal is intense (likely due to multiple copies of the transgene) in sparsely labeled neurons. The intensity of GFP expression in basket interneurons transfected by our AAV-LS_1_L-GFP virus at 15 days post-injection (dpi) was comparable to that of Thy1-GFP labeled pyramidal neurons in young adult mice [29]: GFP intensity at the somata of brightly labeled AAV-LS_1_L-GFP basket cells (GFP_AAV:Pv_) was 1.48+/−0.10 fold higher compared to that of Thy1-GFP labeled pyramidal neurons (GFP_Thy1_) (Fig. 1f). Furthermore, we found that the intensity of AAV-driven GFP expression was similar between pyramidal neurons in Emx-cre mice and basket interneurons in Pv-cre mice (GFP_AAV:Emx_/GFP_Thy1_ = 1.27+/−0.19, Fig. 1f). Thus AAV-LS_1_L-GFP expression was independent of cell type and Cre driver. Using the same method, we also measured GFP expression levels in the Z/EG indicator mice [22] bred with Pv-cre mice (Pv-cre::Z/EG). GFP intensity was more than 50-times weaker in basket interneurons in Pv-cre:: Z/EG mice compared to pyramidal neurons in Thy1-GFP mice (GFP_Pv-cre:Z/EG_/GFP_Thy1_ intensity = 0.016+/−0.10). GFP expression level in Pv-cre::Z/EG mice was too low to image axons or dendrites, and labeling efficiency was low (Fig. S2).

### Expression of AAV-LSL-GFP is stable for months

We characterized the time course and stability of GFP expression after AAV transfection in Pv-cre mice. GFP signal was prominent as early as 6 days post-injection. Expression levels increased further at 15 days post-injection, and remained strong for at least 60 days. Expression levels of individual AAV-transfected cells in Pv-cre mice were heterogeneous, even close to the injection site, as evident in the histogram plots in Figure 2a. Variable expression levels could be the result of cell-to-cell variation in the number of stabilized AAV genomes [30] and/or the number of Cre-activated AAV genomes. In contrast to Pv-cre mice, control wildtype mice injected with AAV-LS_1_L-GFP showed little GFP signal (Fig. 2a). In some cases, a few faint GFP labeled cells were detected, often in the infragranule layers, suggesting that there was a very slight leakiness of the STOP cassette in AAV-LS_1_L-GFP; however, both the incidence and the level of such leaky expression were exceedingly low (Fig. 2a).

AAV is considered to be non-pathogenic even after long-term transfection [15], [18], [19]. In Pv-cre mice several weeks after AAV-LSL-GFP transfection, the highly exuberant basket axons and characteristic perisomatic boutons suggest that AAV has no detectable adverse effects on the morphology of interneurons. To examine if there was any adverse effects on intrinsic physiological properties of basket interneurons, acute cortical slices were prepared 18–20 days after transfection, and GFP-positive cells were analyzed by whole cell recording. AAV-transfected basket interneurons maintained their characteristic non-accommodating, fast-spiking phenotype [31] in response to depolarizing current injection (Fig. 2b). As expected for PV^+^ basket interneurons, these cells had low input resistance (115.55+/−32.00 mΩ (n = 3 cells); their spikes were narrow, with a spike half-width of 0.82+/−0.06 ms at 26 degrees Celsius and a large spike after-hyperpolarizations (18.55+/−4.07 mV). These results indicate that the intrinsic physiological properties of interneurons were preserved after AAV transfection. Together, our results suggest that gene delivery by Cre-activated AAV is suitable for long term morphological, physiological, and behavioral studies.

### Functional demonstration that the Cre-activated AAV method is cell-type specific

We further demonstrated the versatility of the Cre-activated AAV method by expressing channelrhodopsin-2 (ChR2; [32], [33]), a rapidly gated light-sensitive cation channel capable of depolarizing neurons to spike threshold in response to brief pulses of blue light. A ChR2-mCherry fusion protein [32] was used to create AAV-LS_2_L-ChR2mCherry, and this viral vector was injected into 2-week old Pv-cre mice. An electrophysiological assay in acute slices prepared 1–2 weeks after injection was used to functionally test for cell-type specific expression of ChR2.

First, we demonstrated that AAV-transfected neurons were directly light sensitive (Fig. 3a–c). Transfected neurons were targeted for either cell-attached or whole-cell recording by visualizing mCherry fluorescence (Fig. 3a). Light-driven action potentials (spikes) were reliably evoked in ChR2mCherry+ neurons in response to brief exposure to blue light. The number of spikes increased with longer duration light exposure; and prolonged light stimulation evoked a pattern of spiking that is consistent with PV+ interneuron “stuttering” pattern of spiking in response to prolonged depolarizing current injection (Fig. 3b; [31]). Light-evoked instantaneous firing rates reached up to 260 Hz. In response to 25 ms duration light pulses, the average latency to spike was 4.83+/−1.13 ms, and ranged from 2.3 to 9.4 ms (n = 6). To confirm that the depolarizing effects of light were direct, whole-cell recordings of ChR2mCherry+ neurons were made in response to light stimulation that was set to be sub-threshold for spike generation, in the presence and absence of TTX, a blocker of action potentials (Fig. 3c). As expected, light-evoked depolarizing current was maintained in the presence of TTX, excluding the possibility that depolarization was di-synaptically driven. The average latency to current on-set before TTX application was 0.73+/−0.19, and was not altered in the presence of TTX, 0.77+/−0.17 ms (n = 3).

Next, whole-cell recordings were made in ChR2mCherry-negative pyramidal cells post-synaptic to transfected neurons, in areas of dense mCherry labeling (Fig. 3e). The following assay was used to demonstrate that ChR2mCherry expression was restricted to PV+ interneurons: post-synaptic responses were recorded at the reversal potential for AMPA/NMDA-mediated current (0 mV), a holding potential at which inhibitory current is detected and isolated from excitatory current, and then post-synaptic responses were recorded at the reversal potential for GABA-mediated current (−55 mV), a holding potential at which excitatory current is detected and isolated from inhibitory current. In response to a 25 ms duration light pulse, inhibitory current was recorded at a holding potential of 0 mV (Fig. 3e). Inhibitory responses were detected in a total of 14 neurons, the average response amplitude was 231+/−53.5 pA (range: 649.5–38.5 pA). The average latency to current on-set was 6.63+/−0.55 ms, therefore considering that the average latency to spike in ChR2mCherry+ neurons was 4.83 ms, the post-synaptic response latency is consistent with monosynaptic activation of the current. Of the 14 inhibitory-responsive neurons identified, no excitatory post-synaptic current was detected, nor was any excitatory post-synaptic current detected in any of the other neurons recorded (n = 7) that were negative for an inhibitory response. To confirm that if present, excitatory current would be detected under these recording conditions, electrical stimulation was used to evoke both inhibitory and excitatory current (Fig. 3d), furthermore, both inhibitory and excitatory spontaneous synaptic events were detected (Fig. 3e, inset). The above data demonstrate that expression of ChR2 was specific to inhibitory neurons.

### In-vivo imaging of PV+ inhibitory interneuron axon structural dynamics

**Figure 4:**
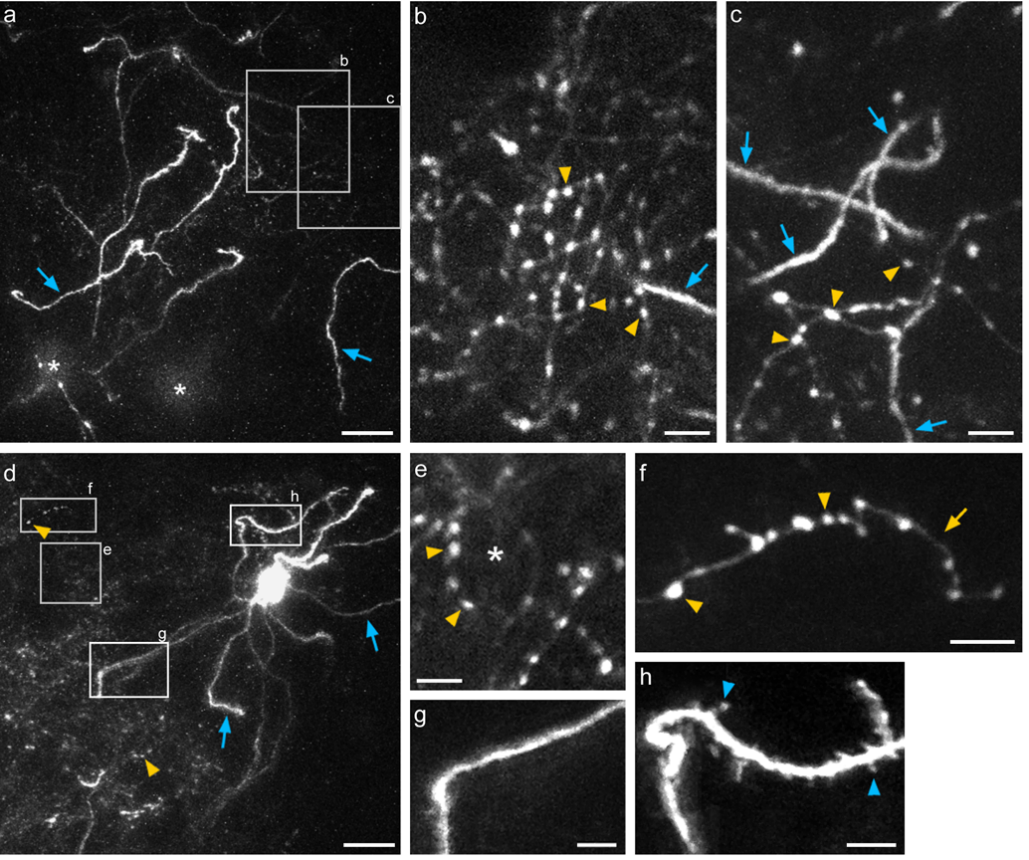
*In-vivo* 2-photon imaging of GFP-labeled basket interneurons in Pv-cre mice. (a) Low-magnification projection of a z-series starting at ∼20 µm below the pia surface to a depth of ∼120 µm, from a densely labeled area. The asterisks indicate the tops of two cell bodies. Numerous dendritic branches are indicated by blue arrows. (b–c) Higher magnification z-projections of regions highlighted in (a) at two different depths: 85–90 µm (b) and 65–75 µm (c) below the pia. Note the smooth, aspiny dendrites (blue arrows), and dense cluster of boutons of varying size (yellow arrowheads). (d) Projection of a z-series 60–170 µm below the pia from a sparsely labeled area, showing an isolated GFP-labeled basket interneuron. Dendrites (blue arrows) could be traced back to soma, and axonal boutons (yellow arrowheads) appear as a cloudy signal at this magnification. (e–f) Examples of axon morphology magnified from areas indicated by gray boxes in (d). (e) shows an axonal basket-like structure (asterisk), and (f) shows a well-isolated axon branch (yellow arrow) with distinct boutons (yellow arrowheads). (g–h) dendrite structures from areas indicated in (d). Dendrites of the cell were largely aspiny (g), though occasionally small protrusions were visible on some dendritic branches (h). Scale bars a,d: 20 µm; b–c, e–h: 5 µm.

We performed in-vivo, long-term imaging of basket interneurons by two-photon microscopy in the neocortex of Pv-cre mice (n = 3) injected with AAV-LS_1_L-GFP. Mice were implanted with glass windows directly over the cortex. GFP expression was bright enough to allow reliable imaging of dendrites, axons, and putative presynaptic boutons visualized as distinct swellings along the axon (Fig. 4). In areas of dense labeling, plexuses of basket cell dendrites and axons intertwined, and clusters of “basket-like” boutons were routinely observed (Fig. 4a–c, see Movie S1 and Movie S2 to view high-magnification z-series stacks). At the edge of the injection site in all 3 experimental animals, where there was sparse labeling, dendrites of individual cells could be easily traced back to the soma (Fig. 4d, see Movie S3 to view 3-D rotation). We found that dendrites were primarily aspiny and smooth (Fig. 4b,c,g), although in some cases dendritic protrusions were apparent (Fig. 4h, see Movie S4 to view z-series stack). The density and size of dendritic protrusions seen here is similar to previous characterizations of cortical fast-spiking basket cells [34]. Well-isolated axon branches and boutons were frequently visible (Fig. 4e,f).

**Figure 5:**
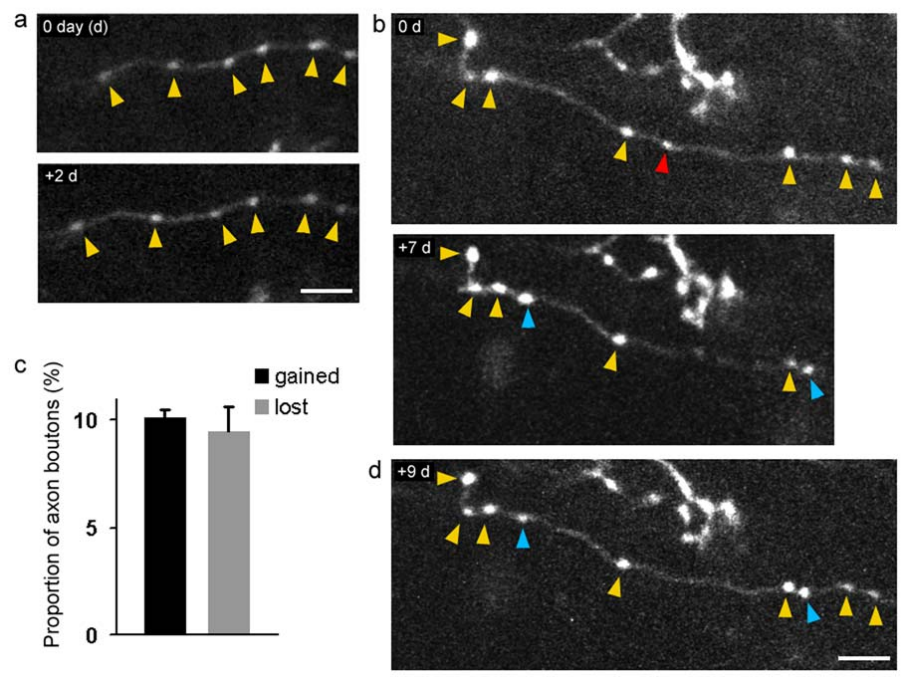
Chronic 2-photon imaging of structural dynamics of basket interneurons in-vivo. (a) Example images of the morphology of an axon branch and boutons in which both were stable over the course of two days. (b) One-week imaging interval in which the axon branch and boutons were largely stable (yellow arrowheads), additions (blue arrowheads) and subtractions (red arrowheads) on the axon shaft were observed. Note, two far right boutons are not shown for day 7. (c) Proportion of axon bouton additions and subtractions (total number of boutons examined = 183, from three animals). (d) Example in which newly formed boutons persistented for at least two days (blue arrowheads). Scale bars: 5 µm.

A major advantage of sparse labeling is that it allows reliable and repeated imaging of dendritic and axonal structures of the same neuron [27]. In the GIN transgenic line [35], GFP expression level is sufficient for in vivo imaging of somatostatin-positive interneurons, but the dense labeling of overlapping dendritic arbors prevented tracking of dendritic branches longer than ∼40 microns and dense labeling of axonal structures made it difficult to locate the same region for repeated measures (S.K., unpublished data). In the neocortex of AAV-LS_1_L-GFP injected Pv-cre mice in areas of sparse labeling, we were able to repeatedly image long stretches of the same dendritic branch (not shown) and axonal structures (Fig. 5) over the course of weeks. Furthermore, dendritic branches were easily traced back to the soma (Fig. 4).

We monitored the axon dynamics of post-adolescent, young adult mice (3 months old) over the course of one week. Axon boutons were found to be fairly stable over 2-day intervals (Fig 5a), and no major changes in axon morphology were observed. Longer-term imaging revealed axon boutons are dynamic in young adult mice, although the majority of boutons were stable over the course of one week. Axon bouton additions and subtractions were readily observed on the axonal shafts (Fig. 5b–c). The number of additions and subtractions was balanced (average number of additions per animal (n = 3): 10.10+/−0.41%; subtractions: 9.47+/−1.18%, a total of 183 boutons were examined). The average distance between boutons was 5.5 µm, consistent with previous reports in fixed tissue [34]. In a few cases it was possible to monitor the persistence of newly formed boutons. We found that these newly formed boutons persisted for the remainder of the imaging experiment (Fig. 5d). These results demonstrate that in vivo, inhibitory interneuron axons are capable of structurally remodeling in the post-adolescent brain.

## Discussion

With increasingly comprehensive knowledge of gene expression patterns in the brain [10], it is apparent that gene expression profiles correlate to known anatomically and physiologically defined neuron classes [36]. Cell-type specific transcription promoters represent crucial tools to systematically establish “genetic access” to cell types, in part achieved by the generation of Cre drivers [12]. Here we improve this strategy by incorporating viral-mediated gene expression into the Cre/loxP binary system. By combining the strength of Cre drivers and viral-mediated gene transfer, we developed a versatile and cost-effective method to label and manipulate specific classes of neurons in mouse brain. Two important advances were achieved: 1) high-level gene expression suitable for live imaging of synaptic structures of specific cell types in vivo; 2) light activation of specific neuron types via expression of channelrhodopsin. The method is also versatile in terms of labeling efficiency. We demonstrated that labeling efficiencies of >95% can be achieved. For applications requiring sparse labeling, efficiency can be reduced by decreasing the volume of virus injected, and by directing imaging studies to the periphery of viral transfection. This is in contrast to transgenic lines, in which efficiency is fixed (ie, compare Fig. 1 with Fig. S2). Furthermore, viral injection allows gene delivery not only to a restricted cell type (defined by the transcriptional promoter used to drive Cre) but also to a particular brain region and developmental stage, an advantage in behavioral studies. Finally, viral production is more cost- and time-efficient compared to mouse genetic engineering. It should be noted though that AAV has a limited packaging capacity of roughly 5 kb of exogenous DNA [37].

Cortical GABA inhibitory interneurons are crucial for all aspects of neural circuit operation. Furthermore, structural axonal remodeling of inhibitory neurons has been identified as a potential cellular mechanism underlying the fine-tuning of cortical circuitry during experience-development and adult learning. For example, both whisker [6] and visual [5] deprivation decreases the number of inhibitory synapses contacting pyramidal cells, while whisker stimulation leads to a persistent increase in the number of presynaptic inhibitory boutons targeting pyramidal spines [4]. A recent study suggests that dendritic remodeling may be more prominent in cortical GABAergic interneurons compared to pyramidal neurons [38]. However, due to the heterogeneity of GABAergic cell types and the lack of a high resolution labeling method, the structural dynamics of defined classes of inhibitory cortical neurons and synapses has not been studied in the intact brain. By 2-photon imaging of Cre-activated viral gene expression in a specific cell type, we observed, for the first time, in vivo structural dynamics of Pv+ basket interneurons. We found that although putative presynaptic boutons from Pv+ basket cells are largely stable, they do remodel even in young adults.

### General Applications

Because the construction of conditional AAV vectors is straightforward and economic, a variety of applications can be readily implemented. First, intense labeling of specific cell types can be used to trace long-range axon projections [19] and also to purify cell types for analysis of gene expression profiles [9]. Furthermore, subcellular-targeted versions such as a synaptophysin-YFP fusion protein, will allow high resolution imaging of defined neuronal structures in vivo. Second, expression of RFP or channelrhodopsin in specific cell types will facilitate cell type identification in physiology and imaging experiments. Two major techniques used to study mammalian neuronal network activity in vivo, tetrode recording of action potentials and bulk-loaded calcium imaging [39], suffer from the inherent problem of cell type heterogeneity - the cell type identity of the recorded or imaged neuron is extremely difficult to determine. This is true even at the most basic level of distinguishing excitatory vs inhibitory neurons. The method we developed in this study may help alleviate this problem. AAV-LSL-RFP can be used to pre-label a specific cell-type in calcium imaging experiments in vivo, so that the calcium dynamics of a known cortical cell-type can be tracked. In addition, AAV-LSL-ChR2 could be used to identify cell types during tetrode recording. This could be achieved by comparing and matching the spike waveform of isolated units to light-evoked spike waveforms of a known, genetically defined cell-type. Third, conditional AAVs expressing channelrhodopsin or halorhodopsin could be used to activate or inhibit specific populations of cells at precise temporal epochs during a behavioral task to test the relevance of the specific cell-type in circuit function. In addition, dominant negative or shRNA constructs could be introduced via a conditional AAV vector to suppress gene function, thereby modulating the activity of specific cell types in specific brain regions in vivo.

Recognizing the importance of cell-type based approach, the NIH Neuroscience Blueprint Project has funded efforts to systematically generate Cre-expressing mouse lines (http://grants.nih.gov/grants/guide/rfa-files/RFA-MH-06-007.html). Cre-activated viral gene expression increases the usefulness and impact of Cre mouse lines, and represents a significant technical advance in studying neural circuit organization and function. In addition to Cre/loxP, Flp/frt is another well-established recombination system in mice [40]. A combination of the two systems should allow simultaneous labeling and manipulation of two cell types.

## Methods

Mice were treated in accordance with Cold Spring Harbor guidelines on animal husbandry and care/welfare.

### Generation of LSL.XFP AAV vectors

To generate a STOP cassette suitable for AAV vectors, we reduced the size of the STOP cassette in the Z/EG construct [22](a gift from C. Lobe) from 4959 bp to 1788 bp. This was achieved by deleting a large portion of the βgeo fusion gene: the XbaI-SphI fragment of βgeo, containing the upstream loxP site and much of the βgeo fusion gene, was replaced with a 657bp PCR fragment containing the upstream loxP site and a short N-terminal fragment of the lacZ gene. The PCR primers were: 5′-GTTCGGCTTCTGGCGTGTG ACC-3′, 5′TTTGGGCATGCGAAACGCCGAGTTAACG CCATC-3′. The resulting “Δβgeo/EG” construct contains a 2.8 kb EcoRI-NotI fragment containing, from 5′ to 3′, loxP, Δβgeo, 3xpolyA, loxP, and GFP cDNA. This 2.8 kb fragment was transferred to an ITR-containing AAV-MCS plasmid (Stratagene) at the HincII site, downstream of a CMV promoter and a beta-globulin intron. The final insert size was 4682 bp in this AAV-LS_1_L-GFP vector. 1.5 mg of the AAV-LS_1_L-GFP vector was purified by maxiprep (Qiagen) and packaged into AAV by University of Pennsylvania Vector Core Facility.

To facilitate the replacement of GFP with any transgene gene of interest (∼≤2 kb), a LoxP-STOP-LoxP shuttle plasmid was designed. We used a short STOP cassette (1298 bp; LS_2_L) consisting simply of the neomycin gene followed by 2 HSV polyA signals to terminate transcription, flanked by 2 loxP sites. This LS_2_L cassette was designed by Dr. Michael Lazarus in the laboratory of Dr. Clif Saper (Harvard University, unpublished), which we confirmed to be sufficient to render GFP expression conditional upon Cre-mediated recombination (Fig. S1). The neomycin STOP cassette was subcloned into the AAV-MCS plasmid. Most of the original AAV-MCS multiple-cloning site (MCS) was left intact, located immediately downstream of the STOP cassette, thus facilitating inserting a gene of interest for future studies. The first ITR to the end of the STOP cassette was 2660 bp in length. Considering that AAV has a ∼5 kb packing capacity [37], a transgene of about 2 kb or less is suitable for this vector. Removal of a large growth hormone polyA signal present in our shuttle vector could increase gene insert size to 2.5 kb. To generate the AAV-LS_2_L-RFP construct, dsREDexpress (Clontech) was subcloned into the LS_2_L shuttle plasmid at restriction sites EcoRV and HindII. To generate the AAV-LS_2_L-ChR2mcherry construct, ChR2mcherry [32] was subcloned into the LS_2_L shuttle plasmid (D. Kvitsiani, unpublished). AAV vectors were prepared as described above.

### Viral Injection

Young adult mice (1.5–3 months old, unless otherwise noted) were anesthetized with an intraperitoneal injection of ketamine/xylazine mixture (0.13 mg/g, 0.01 mg/g body weight). A small hole in the skull was made using a dental drill (Henry Schein), 0.5 mm posterior from Bregma, and 1.5 mm from the midline. The dura was slightly punctured and virus was delivered by pressure injection using a glass micropipette (tip size of roughly 10 µm) attached to a Picospritzer (General Valve). The glass micropipette was lowered to 0.5 mm below the pia surface. To inject virus into the brain, 40 air puffs were delivered (25 psi, 10 ms duration) at a frequency of 0.2–0.4 Hz, the pipette was then retracted 50 microns towards the surface, and pressure injection repeated. This sequence was repeated until the pipette tip reached a depth of −250 microns below the surface. The pipette was then held in place for approximately 5 minutes before completely retracting out of the brain.

### Immunocytochemistry and co-localization quantification

Mice were anesthetized (sodium pentobarbitone; 6 mg/100 g body weight) 15 days post-injection and perfused transcardially with 4% paraformaldehyde in phosphate buffer, pH 7.4. Eighty micrometer- thick coronal sections were cut using a vibratome (Leica VT100). Brain sections were blocked in 10% NGS and 1% Triton. Slices were then incubated overnight at 4°C in 10% NGS, 0.1% Triton and primary antibody NeuN (mouse monoclonal, 1∶400 dilution; Chemicon), PV (mouse monoclonal, 1∶1000; Sigma), or LacZ (mouse monoclonal, 1∶200, Promega). Sections were then incubated with Alexa594-conjugated goat IgG (1∶400; Molecular Probes), mounted on slides with VectaBond mounting media (Vector Laboratories) and coverslipped (glass thickness: #0). To quantify labeling efficiency, confocal images were acquired, and individual cells were identified independently for each of the two fluorescent channels by hand, then subsequently scored for co-localization. Confocal microscopy was performed as described in Chattopadhyaya 2004 [5], using a Zeiss LSM510 microscope and either a 20× objective numerical aperture (NA) 0.5 or a 63× oil objective (NA 1.4). Scans from each channel were collected in multiple track mode to avoid cross-talk between channels.

### Quantification of GFP fluorescence intensity

Brain sections from AAV transfected mice were prepared as described above. Epi-fluorescent images were acquired using an Axioskop FS2 upright microscope (Zeiss) equipped with a 10× objective (NA 0.5, Zeiss), mercury bulb, 0.5 neutral density filter, narrow-band GFP filter set (Chroma), and a cooled charged-coupled devise camera (ORCA-ER; Hamamatsu). Images were digitized at 12-bits, the full intensity range of 0–4096 was used, without saturating the signal. The most densely labeled slice in a series of 80-micron sections was selected (the presumed site of injection) and the intensity of all cell somata in the selected slice was quantified. To calculate intensity of individual cell soma, region-of-interests (ROIs) were placed over all cell somata in the slice and the nine most intense pixels for a given cell was averaged, background-subtracted, and a threshold of 2 times the difference between the background maximum and minimum pixel intensity was applied, using ImageJ software. Background was calculated as the mean intensity of a 200 micron^2^ area in the non-injected contralateral cortex. In the case of transgenic mice, background was calculated as the mean intensity of 10 different 20 micron^2^ areas of non-labeled cortex. Exposure time was 75 ms for the data quantified in Figure 2, and 50 ms for the data quantified in Figure 1. Intensity of both AAV-labeled and Thy1-GFP labeled cells within a single brain slice was highly variable. We were interested in comparing the most intensely labeled Thy1-GFP cells in transgenic mice with our AAV-mediated labeling method. Therefore, the intensity of 25–35 cell somata was quantified for each condition, and the average of the 15 most intense cells was reported in Figure 1f. All reported error estimates were calculated as standard error.

### Electrophysiology

GFP-expressing neurons transfected with AAV.LS_1_L.GFP and mCherry-expressing neurons transfected with AAV.LS_2_L.ChR2mCherry were visualized using the upright microscope described above and cells were patched using a 63× objective (NA 0.95) under near-infrared differential interference contrast optics. A Texas Red filter set (Chroma) was used in the case of mCherry. Whole-cell current-clamp recordings were made from slices submerged in ACSF (in mM): 126 NaCl, 2.5 KCl, 1.25 NaH2PO4, 1 MgSO4, 2 CaCl2, 10 D(+)- glucose, and 25 NaCHO3; continuously bubbled with 95%O2/5%CO2, perfused at a rate of 2–3 ml/min (26°C). Unless noted, pipettes were filled with intracellular solution (in mM): 135 K-gluconate, 4.3 KCl, 2 NaCl, 10 HEPES, 0.5 EGTA, 4 MgATP, 20 phosphocreatine(Na), and 0.3 NaGTP, pH 7.3, 300 mOsm having a resistance of 2–4 MΩ. Data were acquired at 5–10 kHz and low-pass filtered at 2 kHz. Electrophysiological signals were processed and controlled by a Multiclamp 700A amplifier, Digidata 1322 analog-to-digital converter, and Clampex9 software (Molecular Devices). All reported error estimates were calculated as standard error.

Light stimulation was delivered using full-field epi-fluorescence through the narrow band GFP filter set. The timing of the light stimulus was controlled via a shutter (model VS25, UniBlitz) and triggered using Clampex9 software. The duration of light delivery was calibrated using a light meter and reported as the duration between the onset and offset of >80% maximum light intensity. For post-synaptic recording, pipettes were filled with (in mM): 120 Cs-gluconate, 8 KCl, 10 HEPES, 10 EGTA, 10 QX-314. The reversal potential of GABA-mediated chloride current for this internal solution was empirically measured in response to electrical field stimulation in the presence of 20 µM CNQX and 100 µM APV.

### In vivo 2-photon imaging

Chronic windows were made following the protocol described in Holtmaat (2004) [41], 2–3 weeks after the injection of AAV-LS_1_L-GFP. Animals were anesthetized with a ketamine/xylazine mixture (0.13 mg/g, 0.10 mg/g body weight) and given a small does of dexamethasone (0.02 ml at 4mg/ml) to reduce inflammation. The skin over the skull was removed and lidocaine was topically applied. An area of skull (approximately 1 mm in diameter) just posterior to the previous viral injection was removed using a dental drill (Henry Schein), leaving the dura intact. A glass coverslip (EM Sciences, #1, 5 mm round) was then coated with slightly warmed agarose (Type III-A, 1% in HEPES buffered ACSF), gently placed over the craniotomy, and sealed with dental acrylic.

On the day of imaging, mice were anesthetized with a ketamine/xylazine mixture (0.13 mg/g, 0.10 mg/g body weight) and mounted in a fixed position below the microscope objective. In-vivo images of GFP-labeled cells were acquired with a custom made 2-photon laser scanning microscope [13], controlled by ScanImage software [42]. The light source was a Ti:Sapphire laser (Mira 900, Coherent) running at λ 910 nm, powered by a 10-watt pump laser (Verdi V-10, Coherent). Light was focused with scan lens and an objective (40× water-immersion objective, NA 0.8) from Zeiss. Signal was detected with a photomultiplier tube (Hamamatsu). Image stacks were collected in 1micron steps, at a resolution of 0.36–0.075 microns per pixel.

Axon boutons were counted with the aid of ImageJ and Matlab software. Raw images were median filtered (2.0 pixels) prior to quantification. Axons were traced by hand in ImageJ and intensity profile plots (1-pixel thickness) were imported into MatLab. The approximate derivative of the intensity profile plot and the standard deviation of the derivative was calculated for each axon segment, and the presence of a bouton was scored if the derivative value was greater than the positive standard deviation for at least 3 consecutive points, and crossed the negative standard deviation without fluctuating across the zero line.

## Supporting Information

Figure S1 STOP2 confers conditional expression. (a) Epi-fluorescence image of a Pv-cre mouse injected with an AAV vector containing the short Neo-2xpA STOP cassette (LS2L) followed by the human RenillaGFP gene (AAV-LS2L-hrGFP, gift of C. Saper). (b–c) Confocal images of a wildtype control mouse co-injected with AAV-LS2L-hrGFP (b) and a generic AAV-RFP (c), at a 3∶1 ratio. (d) Co-localization (white arrowheads) of GFP and parvalbumin (PV) in neocortical basket interneurons in neocortex of a Pv-cre mouse injected with AAV-LS2L-hrGFP; scale bar, 20 microns. Far right, high resolution image of basket cell axons with “basket-like” terminal branches and boutons (yellow arrowheads) around pyramidal cell somata (labeled with NeuN immunofluorescence) characteristic to PV+ interneurons; scale bar, 5 microns.
(3.73 MB TIF)

Figure S2 Low-level GFP expression in Pv-cre::Z/EG mouse. Epi-fluorescent image of young adult Pv-cre::Z/EG mouse using a 1 sec exposure time (13.3-times longer than that used in Fig. 2a) and a compressed look-up table. Cells (arrowheads) were not visible using the same acquisition parameters as in Fig. 2a. Neither axon nor dendritic branches were readily visible, though occasionally a dendritic branch trunk close to the soma was noted (arrow). Scale bar, 100 microns.
(0.45 MB TIF)

Figure S3 60 days post-injection. Image of young adult Pv-cre mouse injected with AAV-LS1L-GFP using same parameters and look-up table as in Figure 2a; scale bar, 100 microns.
(0.38 MB TIF)

Movie S1 z-series stack from which Figure 4b was taken.
(1.10 MB MOV)

Movie S2 z-series stack from which Figure 4c was taken.
(4.61 MB MOV)

Movie S3 3D rotation of cell shown in Figure 4d.
(1.36 MB MOV)

Movie S4 z-series stack from which Figure 4h was taken.
(1.65 MB MOV)
