# Supplementary figures and images for: High-Resolution Labeling and Functional Manipulation of Specific Neuron Types in Mouse Brain by Cre-Activated Viral Gene Expression
Source: PLoS One. 2008 Apr 16;3(4):e2005. doi: 10.1371/journal.pone.0002005 (PMC2289876; doi:10.1371/journal.pone.0002005)

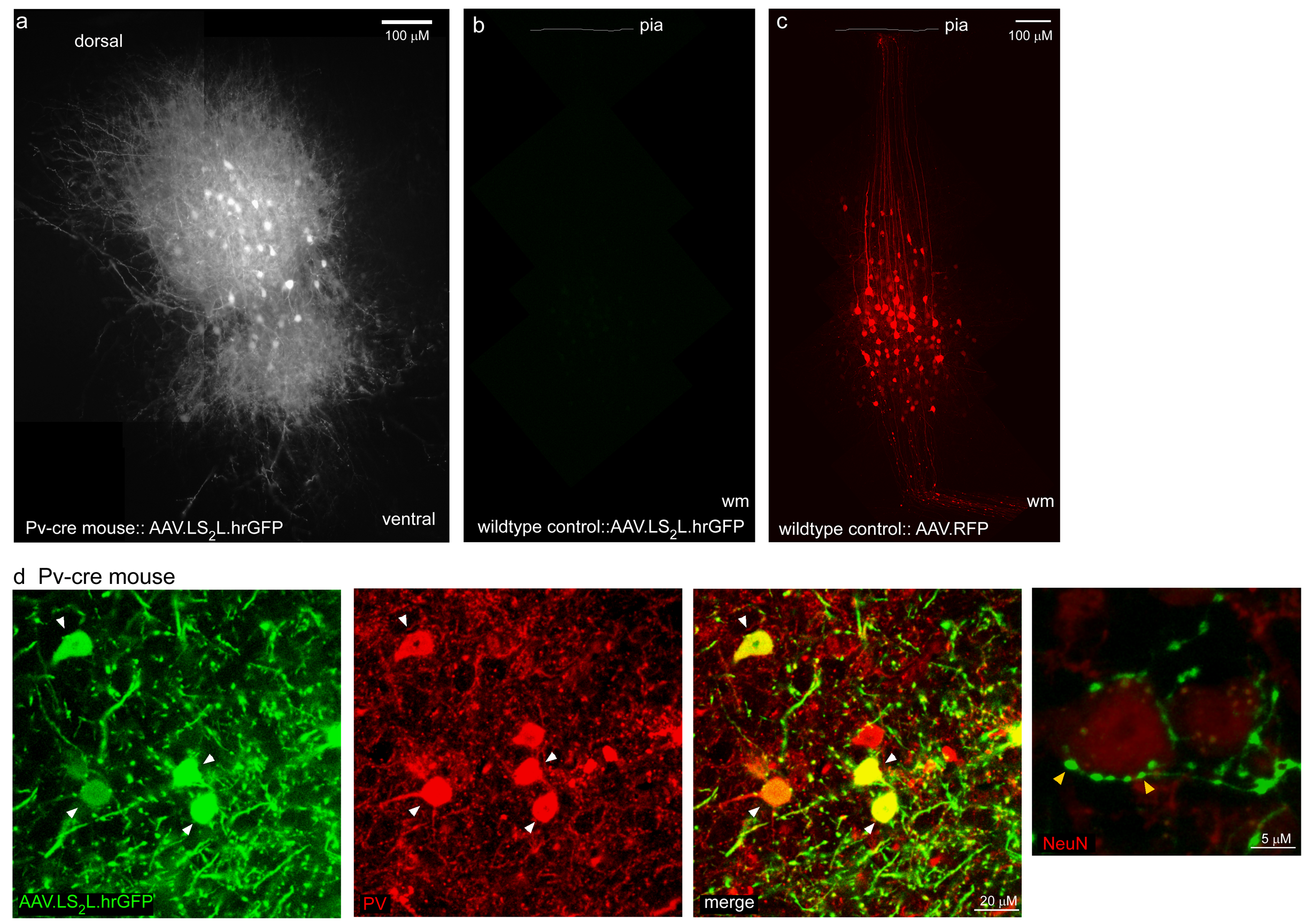

Supplement: Figure S1 — STOP2 confers conditional expression. (a) Epi-fluorescence image of a Pv-cre mouse injected with an AAV vector containing the short Neo-2xpA STOP cassette (LS2L) followed by the human RenillaGFP gene (AAV-LS2L-hrGFP, gift of C. Saper). (b–c) Confocal images of a wildtype control mouse co-injected with AAV-LS2L-hrGFP (b) and a generic AAV-RFP (c), at a 3∶1 ratio. (d) Co-localization (white arrowheads) of GFP and parvalbumin (PV) in neocortical basket interneurons in neocortex of a Pv-cre mouse injected with AAV-LS2L-hrGFP; scale bar, 20 microns. Far right, high resolution image of basket cell axons with “basket-like” terminal branches and boutons (yellow arrowheads) around pyramidal cell somata (labeled with NeuN immunofluorescence) characteristic to PV+ interneurons; scale bar, 5 microns. (3.73 MB TIF) [file pone.0002005.s001.tif]

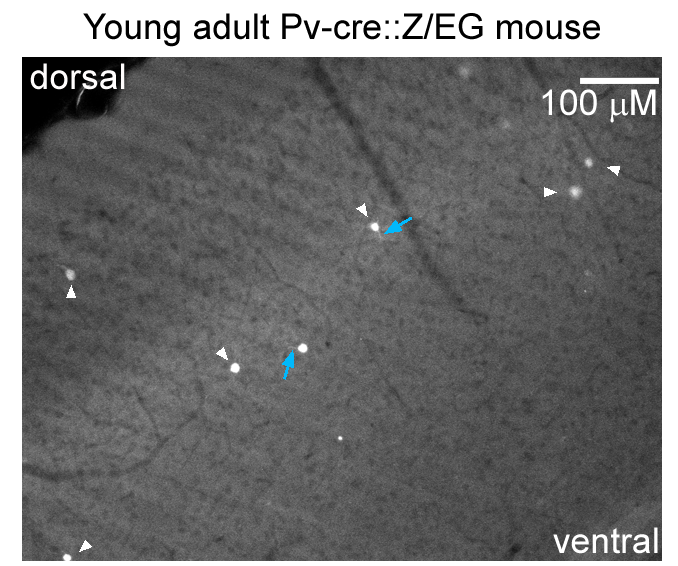

Supplement: Figure S2 — Low-level GFP expression in Pv-cre::Z/EG mouse. Epi-fluorescent image of young adult Pv-cre::Z/EG mouse using a 1 sec exposure time (13.3-times longer than that used in Fig. 2a) and a compressed look-up table. Cells (arrowheads) were not visible using the same acquisition parameters as in Fig. 2a. Neither axon nor dendritic branches were readily visible, though occasionally a dendritic branch trunk close to the soma was noted (arrow). Scale bar, 100 microns. (0.45 MB TIF) [file pone.0002005.s002.tif]

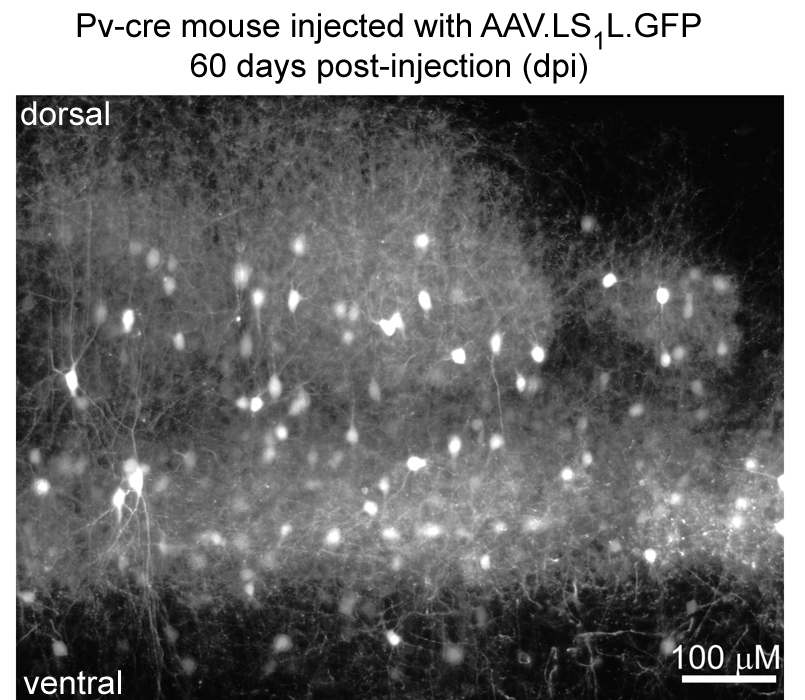

Supplement: Figure S3 — 60 days post-injection. Image of young adult Pv-cre mouse injected with AAV-LS1L-GFP using same parameters and look-up table as in Figure 2a; scale bar, 100 microns. (0.38 MB TIF) [file pone.0002005.s003.tif]
